# Biomimetic Fermentation Reshapes Precursor Pools to Drive Synergistic Roasting Reactions and Enhance Coffee Flavor Complexity

**DOI:** 10.3390/foods15050849

**Published:** 2026-03-03

**Authors:** Shengjie Duan, Lihui Yu, Jinya Dong, Zezhu Du, Shan Liu, Huajie Yin, Yanan Li, Yan Shen, Rongxian Yu, Chaoyi Xue, Yunfei Ge, Li Feng, Xiaocui Du, Yunlan Chen, Ruijuan Yang, Chongye Fang

**Affiliations:** 1 College of Food Science and Technology, Yunnan Agricultural University, Kunming 650201, China; 2 School of Food Science and Technology, Jiangnan University, Wuxi 214122, China; 3 Key Laboratory of Development and Utilization of Food and Medicinal Resources, Ministry of Education, Yunnan Agricultural University, Kunming 650201, China; 4 Yunnan Research Center for Advanced Tea Processing, Kunming 650201, China; 5 College of Agronomy and Biotechnology, Yunnan Agricultural University, Kunming 650201, China; 6 College of Tropical Crops, Yunnan Agricultural University, Pu’er 665000, China

**Keywords:** coffee flavor complexity, in vitro biomimetic fermentation, precursor–product coupling, Maillard reaction thermodynamics, rational flavor design

## Abstract

Deciphering the coupling mechanisms between post-harvest precursor shaping and roasting thermochemistry is pivotal for precise coffee flavor modulation. This study aimed to investigate the regulation mechanisms of in vitro biomimetic fermentation (BF) on the precursor-roasting reaction network. Integrated multi-omics characterization and sensory evaluation reveal that the BF protocol achieves targeted substrate enrichment, notably amplifying free amino acids—particularly leucine and phenylalanine—by 1.89-fold while accumulating lactate and succinate buffering salt systems. This reconfiguration constructs a matrix with superior thermal buffering capacity (ΔpH 0.17), which optimizes the thermal reaction kinetic window during roasting. Consequently, BF drives a 3.08-fold surge in esterification flux, significantly increasing the abundance of key fruity markers such as ethyl acetate and ethyl isovalerate. It also enhances the diversity of Maillard products, specifically elevating nutty-associated alkylpyrazines (e.g., 2,3,5-trimethylpyrazine). Concurrently, BF improves the thermal stability of bioactive compounds, including 5-caffeoylquinic acid (5-CQA) and trigonelline. Multi-scale molecular dynamics and quantum chemical calculations elucidate that BF-derived organic acid–salt complexes exert a ‘pseudo-catalytic effect,’ lowering activation free energy barriers for critical aroma-generating reactions by approximately 8.5 kcal/mol. This study demonstrates high sensory predictability (predictive model *R*^2^ = 0.98) and provides a quantitative theoretical framework to advance coffee processing from empirical observation to rational flavor design.

## 1. Introduction

The formation of coffee flavor represents a temporal coupling between the precursor chemical space established during post-harvest processing and the thermochemical reaction network evolved during roasting [1]. Within this complex food matrix, coffee aroma is orchestrated by a diverse array of volatile compounds. Their sensory contributions are determined not only by concentrations and odor thresholds but also by synergistic or suppressive interactions. Furthermore, the non-volatile matrix modulates the release and perception of these aromas [2]. Concurrently, non-volatile constituents form the flavor backbone—defining acidity, sweetness, bitterness, body, and mouthfeel—which further modulates aroma expression [3]. Consequently, ‘flavor complexity’ should be viewed as a quantifiable phenotype rather than a rhetorical abstraction. Sensorially, it is defined by enhancements in overall quality and richness. Chemically, it involves systematic shifts in key odor-active compounds, underpinned by a consistent coupling between chemical and sensory domains.

Post-harvest fermentation is a critical determinant of coffee quality differentiation. It functions primarily by reshaping substrate pools and aroma precursor reservoirs, which are intimately linked to subsequent roasting reactions. Specifically, microbial metabolism and enzymatic hydrolysis increase the supply of free amino acids and peptides. This alters nitrogen accessibility for Strecker degradation and the Maillard reaction [4]. Similarly, carbohydrate conversion regulates carbonyl donors [5], while lipid hydrolysis provides the foundation for ester formation [6]. Additionally, the accumulation of organic acids and associated pH shifts influence the kinetic windows of thermal reactions [7]. However, spontaneous fermentation is highly variable due to raw material maturity and environmental conditions. These fluctuations make it difficult to attribute chemical differences specifically to the roasting stage. Roasting itself is a non-linear, transient process involving competing pathways like caramelization and lipid oxidation [8]. Without a systematic design, fermentation effects are often obscured by the roasting degree, limiting our understanding of the ‘fermentation–roasting’ coupling mechanism.

Exogenous aroma infusion serves as a critical reference beyond traditional wet or natural fermentation. These sensory profiles bypass the endogenous generation processes inherent to the precursor–thermal reaction network. Consequently, their volatile characteristics and stability across roasting degrees [9] likely differ from established fermentation–roasting trajectories. Integrating such samples into a unified framework provides structured evidence to delineate exogenous contributions from endogenous generation. This approach informs food chemistry imperatives such as authenticity verification and compositional assessment.

The central question of this study is whether controlled fermentation can systematically alter reaction pathways across roasting degrees to shift flavor complexity. We focus on two key processes: first, the formation of esters (fruity and floral notes), which depends on the availability of acid and alcohol substrates during thermal history [10]; second, the Maillard reaction and Strecker degradation (nutty and caramel aromas), which are regulated by precursor ratios, pH, and temperature gradients [11]. Modulating precursor pools, such as acid/alcohol or sugar/amino acid ratios, may redistribute reaction fluxes during roasting. This would manifest as structural shifts in the product spectrum, reconstructed odor activity values (OAVs), and altered aroma wheel contributions [12]. Crucially, these chemical signals should exhibit a consistent coupling trend with Specialty Coffee Association (SCA) cupping scores to verify the “enhancement of complexity.”

To mitigate the challenges of natural fermentation stochasticity, this study employs an in vitro biomimetic fermentation (BF) strategy. This approach achieves reproducible precursor remodeling via programmable microbial synergy under controlled conditions. Benchmarked against washed, natural, and artificially flavored samples, we characterize the co-evolution of physicochemical properties and metabolite profiles across roasting degrees. We utilize a dual-dimensional ‘chemo-sensory’ framework, combining SCA scores [13] with OAVs [14] and informational entropy metrics. Concurrently, non-volatile fingerprints are acquired via UHPLC–MS/MS to elucidate precursor pool remodeling [15]. Finally, we incorporate molecular dynamics (MD) simulations and quantum chemical (QM) calculations to interpret the stability and reaction tendencies of flavor molecules. By integrating experimental and computational data, this study aims to elucidate how BF regulates the fermentation–roasting network and provides a transferable framework for rational flavor design.

## 2. Materials and Methods

### 2.1. Coffee Materials and Experimental Design Across Roasting Degrees

**Scheme 1:**
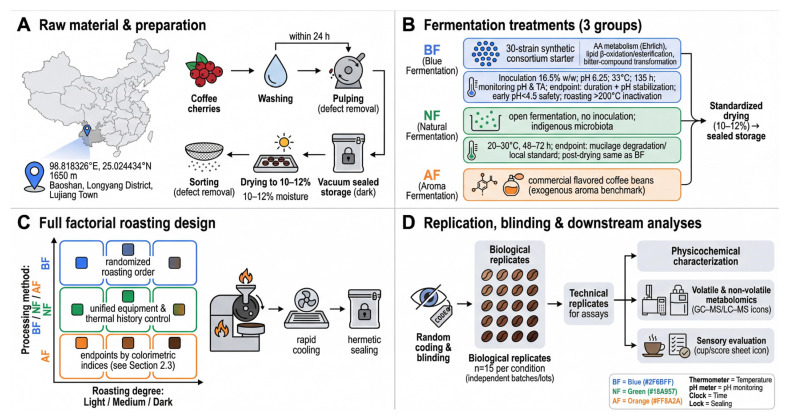
Schematic representation of the experimental design and workflow.

As shown in Scheme 1A,B, raw Material and Preparation: Arabica coffee cherries (*Coffea arabica*) were harvested from the cultivation base in Lujiang Town, Longyang District, Baoshan City, Yunnan Province, China (elevation 1650 m; 98.818326° E, 25.024434° N). To ensure the representativeness and homogeneity of the raw material, a total of approximately 300 kg of cherries were collected and thoroughly mixed using the coning and quartering method before being divided into treatment groups. The sampling time was strategically determined by identifying the peak ripening window, characterized by uniform cherry coloration and a verified average soluble solids content of 18.3 °Brix. Harvested fruits were free from pests and mechanical damage. For each experimental condition, fifteen independent biological replicates (*n* = 15) were established, consisting of separate fermentation batches or sampling lots, to ensure high statistical reliability. Within 24 h post-harvest, cherries were washed, pulped, and sorted to remove defective beans. The resulting green beans were dried under standardized conditions to a safe moisture content range (approximately 10–12% by mass) and then vacuum-sealed and stored in the dark until further use.

Experimental Design and Sampling: Three distinct treatment groups were established: in vitro biomimetic fermentation (BF), natural fermentation control (NF), and artificially flavored (AF) groups, the latter utilizing commercial flavored coffee beans as an exogenous aroma benchmark. Roasting experiments followed a full factorial design of “processing method × roasting degree.” For each treatment group, samples were prepared at light, medium, and dark roasting levels. All roasting was conducted using identical equipment under a unified thermal history control strategy, with roasting endpoints calibrated by colorimetric indices (target ranges and thermal profile parameters are detailed in Section 2.3). The roasting sequence for different treatment groups was randomized. Samples were assigned random codes to ensure blinding during subsequent analyses and sensory evaluations. Independent biological replicates (*n* = 15; representing independent fermentation batches or sample preparation lots) were established for each “treatment × roasting degree” condition, with technical replicates performed for subsequent assays. Roasted samples were rapidly cooled and hermetically sealed for physicochemical characterization, volatile and non-volatile metabolite analysis, and sensory evaluation.

### 2.2. In Vitro Biomimetic Fermentation and Natural Fermentation Protocols

In Vitro Biomimetic Fermentation (BF): The BF process utilized a functionally targeted synthetic microbial consortium as a starter culture. This consortium comprised 30 core strains, selected and assembled to systematically cover key metabolic pathways governing coffee flavor formation, including amino acid metabolism (e.g., the Ehrlich pathway), lipid *β*-oxidation and esterification, and the biodegradation or transformation of specific bitter compounds (e.g., pyrazines). The strain composition and safety assessment are detailed in a concurrent publication [16]. Fermentation was conducted under strictly controlled parameters: inoculation rate (16.5% *w*/*w*, wet bean basis), initial pH (6.25), temperature (33 °C), and duration (135 h). Throughout the process, pH and titratable acidity were continuously monitored to ensure stability and controllability. The fermentation endpoint was determined by a combination of the pre-set duration and physicochemical indicators (e.g., pH stabilization). Upon completion, fermentation was immediately terminated; beans were separated and dried using a standardized protocol to a moisture content of 10–12% and then sealed for storage. Critical safety measures included rapid acidification (pH < 4.5) during the initial phase to suppress non-target microbial growth, and the subsequent high-temperature roasting step (>200 °C) to ensure complete inactivation of the fermentation microbiota.

Natural Fermentation (NF) Control: The NF group followed a typical wet natural fermentation protocol characteristic of the origin. Coffee beans were fermented in an open environment without exogenous microbial inoculation, relying solely on the indigenous microbiota present on the raw material and in the environment. The process occurred at ambient temperature (approx. 20–30 °C) and natural humidity. The fermentation endpoint was determined according to conventional local standards, typically based on duration (e.g., 48–72 h) and/or the degree of mucilage degradation. Post-fermentation, the beans were subjected to post-processing and drying protocols identical to those of the BF group (moisture content 10–12%) to ensure comparability in subsequent roasting and analytical procedures.

### 2.3. Roasting Procedures and Control of Thermal Profiles

As shown in Scheme 1C, roasting was performed using an industrial-grade coffee roaster (Model: SANTOKER-R300; Santoker, Beijing, China) equipped with a programmable hot-air heating system and multi-point temperature sensors. To ensure comparable thermal histories, all roasting operations utilized the same apparatus, which was preheated and stabilized for at least 30 min prior to each session to maintain a constant initial temperature. A dual-control strategy integrating “thermal history–colorimetry” was employed to define light, medium, and dark roasting degrees, each objectively calibrated via reproducible temperature–time profiles and final chromaticity values [17]. Specifically, light roasting targeted an Agtron value of approximately 85, achieved by ramping the bean temperature from 120 °C to 185 °C over a total duration of roughly 8 min with a brief hold; medium roasting targeted an Agtron value of approximately 65, corresponding to a total duration of roughly 10 min with a ramp to 200 °C followed by a hold; and dark roasting targeted an Agtron value of approximately 45, achieved over a total duration of roughly 12 min with a ramp to 215 °C followed by a hold. All roasting profiles were automated via the device’s built-in software, with real-time temperature logging. Each batch consisted of 200 g of green beans, with the hot air flow rate fixed at 1.5 m^3^/h. The roasting endpoint was guided visually by the auditory signal of the ‘first crack’ and definitively determined by the ground bean reflectance value measured using an Agtron G-3000 spectrophotometer (Agtron Inc., Reno, NV, USA) (average of 5 measurements per sample). This ensured stable chromaticity ranges for light (80–90), medium (60–70), and dark (40–50) roasts. Roasted beans were rapidly cooled to room temperature (<3 min) using a high-velocity forced-air cooling tray. Samples were then immediately sealed in nitrogen-flushed bags and stored at −20 °C for no more than 7 days prior to analysis. All roasting sessions were completed within a single day to minimize environmental fluctuations. Samples were randomly coded to eliminate operator bias.

### 2.4. Ultrasound-Assisted Green Extraction of Non-Volatile Metabolites and Bioactive Compounds

Ultrasound-assisted extraction (UAE) was specifically employed for the retrieval of non-volatile compounds and the matrix for bioactive assays [18]. The extraction solvent, a methanol/acetonitrile (1:1, v/v) mixture, was selected based on preliminary solubility trials to maximize the recovery of diverse polar and semi-polar metabolites while ensuring compatibility with the subsequent UHPLC-MS/MS analysis. To ensure experimental consistency and prevent chemical information distortion, a precise sample mass of 5.0 g of roasted coffee beans was ground and subjected to extraction within an identical 30 min post-grinding interval for all batches. Prior to extraction, roasted beans were ground to a uniform particle size of 850 μm using an IKA M20 grinder (IKA-Werke, Staufen, Germany) (60 s grinding time). Extraction was performed using a 10 mm probe-type ultrasonic processor (Model: SCIENTZ-IID; Ningbo Scientz Biotechnology Co., Ltd., Ningbo, China) operating at a frequency of 40 kHz with an actual output power of 400 W (60% amplitude). A pulsed mode (5 s on/5 s off) was applied for a total sonication time of 10 min. Throughout the process, the temperature was strictly maintained at 4 ± 1 °C using an ice-water bath to preserve heat-sensitive bioactive constituents. Following sonication, samples were centrifuged at 12,000 rpm for 15 min at 4 °C. The supernatant was collected and divided into aliquots specifically for UHPLC–MS/MS non-volatile metabolite profiling (Section 2.8) and targeted antioxidant activity assays (Section 2.6). Aliquots were analyzed immediately or stored at −80 °C for no more than one week. This extraction protocol aligns with the principles of green extraction, reducing energy consumption by over 66% and solvent usage by approximately 50% compared to traditional Soxhlet extraction [19].

### 2.5. Physicochemical Analyses

Physicochemical analyses were conducted on the coffee extracts obtained via ultrasound-assisted extraction, with all measurements performed under a controlled ambient temperature of 25 ± 2 °C. The pH was determined using a Sartorius PB-10 pH meter (Sartorius AG, Göttingen, Germany) calibrated with standard buffers at pH 4.00 and 7.00. Total titratable acidity was quantified by titration with 0.1 M NaOH to an endpoint of pH 8.2 and expressed as citric acid equivalents (g/L) [20]. Total dissolved solids (TDS) were measured using a digital refractometer (Model: PAL-1; Atago Co., Ltd., Tokyo, Japan) and expressed as mass concentration (g/mL) [21]. Total polyphenol content was assessed via the Folin–Ciocalteu method, measuring absorbance at 760 nm with gallic acid as the standard; results were expressed as gallic acid equivalents (mg/L), with a standard curve linearity range of 10–200 mg/L (*R*^2^ > 0.999) [22]. Total flavonoid content was determined using the aluminum nitrate colorimetric method, measuring absorbance at 510 nm with rutin as the standard, and expressed as rutin equivalents (mg/L) [23]. Chlorogenic acid content was quantified by HPLC-DAD (Waters 2695, Waters Corporation, Milford, MA, USA, C18 column, mobile phase: acetonitrile/2% acetic acid, detection at 325 nm) using the external standard method [24]. Total free amino acids were measured using the ninhydrin colorimetric method, measuring absorbance at 570 nm with leucine as the standard, and expressed as μmol/g (dry bean basis) [25]. Caffeine content was determined by UV spectrophotometry (Model: UV-2550; Shimadzu Corporation, Kyoto, Japan) at 272 nm and quantified against a caffeine standard curve (linear range 5–100 μg/mL) [26]. All analytical procedures were performed in triplicate, yielding relative standard deviations (RSD) of <5%. Standard curves were verified to be within the linear range, and each analytical batch included blanks and standards for quality control.

### 2.6. Targeted Bioactive Compound Quantification and Antioxidant Assays

Analyses were performed on the coffee extracts obtained via ultrasound-assisted extraction. Antioxidant activity was evaluated using three distinct assays. The FRAP (Ferric Reducing Antioxidant Power) assay [27] utilized the 2,4,6-tripyridyl-s-triazine (TPTZ) reagent; the reaction system (consisting of 2.5 mL TPTZ solution, 2.5 mL FeCl_3_ solution, and 5 mL acetate buffer, pH 3.6) was incubated with the sample at 37 °C in the dark for 10 min, after which absorbance was measured at 593 nm. Results were expressed as μmol Fe^2+^/L. The DPPH radical scavenging assay involved mixing the sample with a 0.1 mM DPPH ethanolic solution, reacting at room temperature in the dark for 30 min, and measuring absorbance at 517 nm to calculate the radical scavenging rate (%), with results expressed as μmol Trolox/L [28]. The ORAC (Oxygen Radical Absorbance Capacity) assay employed fluorescein as the fluorescent probe (70 nM, pH 7.4) [29] and AAPH (15 mM) as the radical initiator; fluorescence decay was monitored at an excitation of 485 nm and emission of 528 nm (readings every 2 min for 90 min), with results expressed as μmol Trolox/L.

Bioactive compounds were quantified via HPLC-DAD. Chlorogenic acid (detection at 325 nm) and caffeine (detection at 272 nm) were quantified using the external standard method [30]. Chromatographic conditions were as follows: Waters 2695 HPLC system, C18 column (250 mm × 4.6 mm, 5 μm), mobile phase consisting of acetonitrile and 2% acetic acid solution (gradient elution: 0–15 min, 5–25% acetonitrile; 15–25 min, 25–60% acetonitrile), column temperature at 30 °C, flow rate at 1.0 mL/min, and injection volume of 10 μL. Total flavonoid content was determined using the aluminum nitrate colorimetric method with rutin as the standard, measuring absorbance at 510 nm. All analyses were performed in triplicate, and results are presented as mean ± standard deviation. Statistical analysis was conducted using SPSS 25.0, employing one-way analysis of variance (ANOVA) followed by Tukey’s HSD post hoc test for multiple comparisons, with *p* < 0.05 considered statistically significant.

### 2.7. Volatile Flavor Compound Profiling by GC–MS

As shown in Scheme 1D, Volatile flavor compounds were determined using solid-phase microextraction (SPME) coupled with gas chromatography-mass spectrometry (GC-MS) [31]. A 5.00 g aliquot of homogenized coffee powder was placed in a 20 mL headspace vial, spiked with 10 μL of 2-octanol (1.00 mg/mL) as an internal standard. The concentration of volatile compounds was calculated as a relative value based on the peak area ratio to 2-octanol, assuming a response factor of 1.0. The average recovery of 2-octanol was determined to be 92.4 ± 3.1%. While we acknowledge that response factors vary by compound, this semi-quantitative approach allows for reliable relative comparisons of flavor shifts across the BF, NF, and AF groups. Extraction was automated using a CTC CombiPAL autosampler. Samples were equilibrated at 90 °C for 5 min prior to extraction. Subsequently, An 85 μm DVB/CAR/PDMS fiber was selected due to its triple-phase coating, which provides optimal recovery for a wide range of coffee volatiles, including low-molecular-weight gases and semi-volatile esters. Samples were equilibrated at 90 °C for 10 min to ensure headspace saturation. Extraction was then performed at 90 °C for 20 min with continuous stirring at 250 rpm using a magnetic stirrer built into the autosampler. The fiber was thermally conditioned at 250 °C for 30 min before the first use.

GC conditions were as follows: a DB-WAX column (60 m × 0.25 mm × 0.25 μm) was used with a temperature program starting at 50 °C (held for 3 min), ramping at 5 °C/min to 100 °C, then at 3 °C/min to 150 °C, and finally at 10 °C/min to 240 °C (held for 10 min). High-purity helium (>99.999%) served as the carrier gas at a constant flow rate of 1.0 mL/min. The inlet was maintained at 240 °C in splitless mode. MS conditions included a transfer line temperature of 280 °C, an ion source temperature of 230 °C, an electron energy of 70 eV, and acquisition in full scan mode (*m*/*z* 35–450). All samples were analyzed in triplicate. Compound identification was confirmed by matching mass spectra against the NIST 2020 library and comparing retention indices (RI). Quantification was performed using the internal standard method (response factor approach). Method validation included determination of limits of detection (LOD), limits of quantification (LOQ), and spike recovery rates (80–120%).

### 2.8. Untargeted Non-Volatile Metabolite Profiling via UHPLC–MS/MS

To complement the targeted analysis in Section 2.6, an untargeted metabolomics approach was employed to capture a broader range of chemical shifts Non-volatile metabolites were analyzed via ultra-high-performance liquid chromatography-tandem mass spectrometry (UHPLC-MS/MS) [32]. The coffee extracts obtained from ultrasound-assisted extraction were filtered through a 0.22 μm nylon membrane prior to injection. Chromatographic separation was achieved on a Waters Acquity UPLC BEH C18 column (2.1 mm × 100 mm, 1.7 μm). The mobile phase consisted of 0.1% formic acid in water (A) and acetonitrile containing 0.1% formic acid (B). The gradient elution profile was as follows: 0–2 min, 5% B; 2–15 min, 5–95% B; 15–18 min, 95% B; 18–20 min, 95–5% B; and 20–22 min, equilibration at 5% B. The column temperature was maintained at 40 °C, with a flow rate of 0.3 mL/min and an injection volume of 2 μL.

Mass spectrometry was performed using an electrospray ionization (ESI) source operating in both positive and negative ion modes. MS parameters were set as follows: capillary voltage, 3.0 kV (positive) or −2.5 kV (negative); source temperature, 150 °C; desolvation temperature, 500 °C; cone gas flow, 150 L/h; and desolvation gas flow, 1000 L/h. Data acquisition utilized a data-dependent acquisition (DDA) mode, consisting of a full scan (*m*/*z* 50–1500) followed by MS/MS scans of the top 8 precursor ions (collision energy ramp: 20–40 eV). Metabolite identification was confirmed by matching accurate mass (error < 5 ppm) and MS/MS fragmentation patterns against the BiotreeDB (V3.0) database. Semi-quantitation was performed using peak area normalization. All samples were analyzed in triplicate.

### 2.9. Molecular Dynamics Simulations

All-atom molecular dynamics (MD) simulations were employed to elucidate the stability and reactive potential of key flavor compounds under simulated roasting conditions at the molecular level. Simulations were executed using the GROMACS 2022.4 software suite, utilizing the OPLS-AA force field combined with the TIP3P water model, a configuration demonstrated to offer superior applicability and accuracy for food systems [33].

System Construction. Based on quantitative data from GC-MS and UHPLC-MS/MS, two representative molecular systems were selected: (1) an esterification-related system comprising acetic acid and ethanol to simulate interactions between ester precursors; and (2) a Maillard reaction-related system comprising glucose and alanine to simulate the initial contact between reducing sugars and amino acids. Molecular structures were retrieved from the PubChem database [34] and geometrically optimized using Gaussian 16 at the B3LYP/6-31G* level. For pyrazines (e.g., 2-methylpyrazine), protonation states were assigned according to the experimental post-fermentation pH (≈6.25). Systems were solvated with TIP3P water [35] in a 3.5 nm × 3.5 nm × 3.5 nm cubic box, with counterions (Na^+^/Cl^−^) added to neutralize the system and maintain physiological ionic strength (0.15 M). To focus on core interactions, the primary simulations included only the selected flavor precursors and solvent, omitting other major coffee matrix components (e.g., polyphenols, complex carbohydrates).

Simulation Parameters. Simulations were conducted in the NPT ensemble at temperatures corresponding to experimental roasting profiles: light roasting (120 °C/393.15 K) and medium roasting (200 °C/473.15 K), with pressure maintained at 1 atm. A V-rescale thermostat and Berendsen barostat were used with a 2 fs time step. Following energy minimization and equilibration (100 ps NVT + 100 ps NPT), 200 ns production runs were performed, saving coordinates every 2 ps. The LINCS algorithm constrained bond lengths, and the PME method handled long-range electrostatics.

Analysis and Correlation. Trajectory analyses included: (1) Conformational stability via Root Mean Square Deviation (RMSD) and Root Mean Square Fluctuation (RMSF); (2) Interaction analysis via intermolecular contact numbers (distance < 0.5 nm), hydrogen bond networks (distance < 0.35 nm, angle > 150°), and contact maps; and (3) Reactivity assessment by calculating distance distributions between key atoms at reactive sites and their probability densities across temperatures. These metrics were used to estimate the probability of reactive contact and were correlated with experimentally observed shifts in product spectra. All simulations were performed in triplicate to ensure statistical reliability, providing molecular-level insights into the “precursor remodeling–reaction network reconstruction” mechanism.

### 2.10. Quantum Chemical Calculations

To elucidate the thermodynamic driving forces and kinetic feasibility of key flavor-related reactions during roasting from an electronic structure perspective, quantum chemical calculations were performed using Gaussian 16. Reaction targets were selected based on GC–MS and UHPLC–MS/MS findings, focusing on key precursor transformations consistent with experimentally observed variations in ester and Maillard product spectra [36]: (1) the esterification of acetic acid and ethanol to form ethyl acetate; and (2) the initial condensation between a reducing sugar and an amino acid, modeled by the formation of a Schiff base (imine) intermediate from open-chain glucose and alanine, representing the early stage of the Maillard reaction. Initial structures for reactants, reactant complexes, products, and transition states (TS) were generated via conformational search and re-optimized from multiple starting configurations to avoid local minima.

Geometry optimizations and frequency calculations were conducted using the M06-2X functional with the 6-31G(d) basis set. Vibrational frequency analysis confirmed that stable points possessed no imaginary frequencies, while transition states exhibited exactly one imaginary frequency [37]. To enhance the reliability of energy barriers and reaction free energies, single-point energy corrections were performed on optimized structures at a higher basis set level (e.g., 6-311+G(d,p)), incorporating zero-point energy and thermal corrections to derive Δ*G^‡^,* Δ*G*, and Δ*H* (at 298.15 K). Given the objective of explaining relative trends under roasting conditions rather than replicating the exact extraction solvent environment, solvation effects were qualitatively addressed using the SMD continuum solvation model with water as the approximate medium (reflecting polar environmental influence on relative barriers). Key conclusions were cross-verified between gas-phase and solvation models for consistency. Where necessary, intrinsic reaction coordinate (IRC) calculations verified that transition states connected the correct reactants and products. Computational results were utilized to support the interpretation of product spectrum differences across fermentation treatments and roasting degrees. By comparing Δ*G^‡^* and Δ*G* values for esterification versus the initial sugar–amino acid condensation, the relative feasibility of these pathways under varying thermal conditions was assessed. These findings were cross-validated against the shifts in ester OAV contributions and Maillard-derived volatile generation trends observed via GC–MS, thereby providing electronic-structure-level support for the hypothesis that “precursor pool remodeling drives reaction network flux redistribution.”

### 2.11. Sensory Evaluation and Flavor Complexity Assessment

Sensory evaluation followed the standard Specialty Coffee Association (SCA) cupping protocol [38,39]. The panel comprised 20 evaluators, including 3 certified Q-graders and 17 trained panelists. Prior to testing, non-Q-graders completed a 16 h calibration training based on the SCA Flavor Wheel and World Coffee Research (WCR) Sensory Lexicon. Cupping sessions were conducted in a standardized sensory laboratory (22 ± 1 °C, 6500 K neutral lighting). Samples were ground uniformly to 850 μm and brewed using fixed parameters and standardized water as per SCA specifications. The coffee was prepared with a water-to-coffee ratio of 150 mL to 8.25 g, with water temperature at 93 °C, Samples were presented blindly with random three-digit codes, and the serving order was randomized using a Williams Latin square design. Evaluators scored 10 attributes—aroma, flavor, acidity, sweetness, aftertaste, body, balance, uniformity, cleanliness, and overall—in 0.25-point increments. The total score was the sum of these attributes. Descriptive aroma/flavor terms were concurrently recorded. Roasting sensory stability was quantified as the inverse of the coefficient of variation (1/CV) of sensory scores across roasting levels.

Flavor complexity was characterized through a combined sensory and chemical approach. Sensorially, descriptive terms were standardized and categorized according to the SCA Flavor Wheel/WCR Lexicon, with synonyms merged. Complexity was quantified via lexical richness (count of unique descriptors) and Shannon information entropy based on term frequency. Chemically, odor activity values (OAVs) were calculated from GC–MS quantitative data and aggregated by aroma wheel categories. Shannon information entropy was calculated using both relative volatile abundance distributions and OAV-weighted distributions. Complexity metrics were statistically analyzed within a “processing method × roasting degree” two-factor framework, with significance set at *p* < 0.05.

### 2.12. Statistical and Multivariate Analyses

All data are presented as mean ± standard deviation. Statistical analyses were performed using R (v4.3.2) and SPSS Statistics (v29.0). Physicochemical indices, antioxidant activities, bioactive components, and sensory scores were evaluated via two-way analysis of variance (ANOVA) to assess the main effects and interactions of processing method and roasting degree. When assumptions of normality and homoscedasticity were met, Tukey’s post hoc test was used for multiple comparisons; otherwise, the Aligned Rank Transform (ART) method was employed for non-parametric two-way analysis.

Prior to multivariate analysis, volatile and non-volatile metabolomics data underwent missing value imputation (half-minimum replacement), log-transformation, and Pareto scaling. Unsupervised Principal Component Analysis (PCA) visualized overall variation and identified outliers. Supervised Partial Least Squares Discriminant Analysis (PLS-DA) elucidated inter-group differences, with model robustness assessed via permutation tests and cross-validation to prevent overfitting. Differential metabolites were screened based on Variable Importance in Projection (VIP > 1.0), statistical significance (FDR-adjusted *p* < 0.05), and fold change (∣log_2_ FC∣ > 1). Metabolic pathway enrichment analysis utilized the KEGG database. Chemo-sensory coupling was examined using correlation analysis (Pearson or Spearman) and PLS regression, constructing association models integrating OAV contributions and descriptive complexity metrics. All tests were two-tailed, with significance defined at *p* < 0.05.

## 3. Results and Discussion

### 3.1. Roasting-Dependent Modulation of Physicochemical Properties

**Figure 1:**
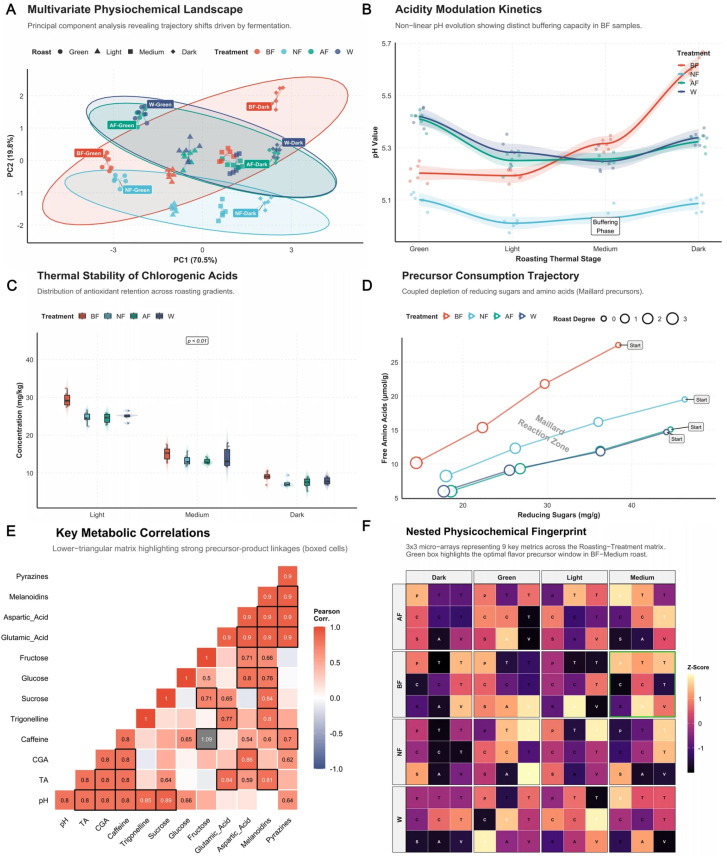
Roasting-dependent modulation of physicochemical fingerprints and thermochemical reaction networks. (**A**) PCA biplot visualizing the chemical space evolution; PC1 (70.5%) reflects the thermal gradient, while PC2 (19.8%) differentiates processing-induced precursor profiles. (**B**) pH trajectories highlighting the superior thermal buffering capacity of the BF matrix (ΔpH = 0.17), likely regulated by fermentation-derived organic acid-salt complexes. (**C**) Thermal stability of chlorogenic acids (CGAs); BF treatment significantly attenuated CGA degradation at medium roast (18.2 ± 1.5 mg/g) compared to NF (12.5 ± 1.1 mg/g, *p* < 0.01). (**D**) Precursor-product coupling plot; the 1.89-fold higher initial TFAAs in BF (28.4 ± 1.2 μmol/g) orchestrated a more robust Maillard flux during the medium-to-dark roasting transition. (**E**) Lower-triangular Pearson correlation matrix; white squares indicate high-confidence correlations (*p* < 0.01), with black frames identifying the synergistic relationship between pH and pyrazine formation. (**F**) Nested physicochemical fingerprint matrix (4 × 4 × 9); the green frame denotes the “Gold Window” in the BF-Medium roast, where precursor retention and flavor-active product generation reach an optimal equilibrium.

The initial chemical space of green coffee beans is predetermined by post-harvest processing, which profoundly dictates their chemical evolutionary trajectory during roasting. Principal Component Analysis (PCA) revealed that the first two principal components explained 90.3% of the total variance [Figure 1A]. Along the PC1 axis, samples exhibited a distinct linear migration corresponding to increasing roasting degree. Conversely, along the PC2 axis, the in vitro biomimetic fermentation (BF) group displayed a projection coordinate significantly distinct from both the natural fermentation (NF) and artificially flavored (AF) groups. This spatial separation quantifies the matrix remodeling effect of the BF protocol, indicating that the intervention of specific synthetic microbial consortia induces a unique, endogenous precursor footprint [40], unlike the superficial aroma modification characterizing the AF group.

Analysis of acidity dynamics [Figure 1B] elucidated the regulatory mechanism of BF treatment on thermally induced pH fluctuations. Throughout the roasting profile, the washed control (W) group experienced drastic pH amplitude shifts (ΔpH ≈ 0.30), whereas the BF group demonstrated superior thermal buffering capacity, narrowing the fluctuation range to 5.18–5.35 (ΔpH = 0.17). This stability is attributed to the specific organic acids (e.g., lactic acid, succinic acid) and their buffering salt systems accumulated via microbial metabolism during biomimetic fermentation [41]. This environment provides a more stable kinetic window for the subsequent Maillard reaction, preventing the excessive catalysis of aroma precursor degradation driven by extreme acidity. Regarding the stability of bioactive components, the thermal degradation of chlorogenic acids (CGA) exhibited significant treatment dependency [Figure 1C]. At the medium roasting stage, CGA content in the BF group (18.2 ± 1.5 mg/g) was significantly higher—by 45.6% (*p* < 0.01)—than that in the NF group (12.5 ± 1.1 mg/g). This suggests that the moderate degradation of the polysaccharide matrix during BF may enhance heat transfer efficiency or mitigate the thermal cleavage of 5-CQA via the pH buffering effect. Concurrently, the consumption trajectory of Maillard reaction precursors [Figure 1D] provided a critical explanation: leveraging an initially high level of free amino acids (28.4 ± 1.2 μmol/g, 1.89-fold that of the washed group), the BF group exhibited a steeper precursor consumption slope (ΔAA/ΔSugar) upon entering the “Maillard reaction zone,” indicating a stronger chemical flux and predicting a higher abundance of nitrogen-containing heterocyclic compounds. The correlation matrix [Figure 1E] further revealed the coupling network among metabolites. Results showed a robust positive correlation between pH and pyrazine generation (*r* > 0.85, *p* < 0.01, marked by white squares), validating the promotional role of the stable pH environment in the BF group on key flavor formation. Finally, the nested fingerprint matrix [Figure 1F] provided panoramic evidence that the BF group achieved an optimal balance of chemical indices within the “medium roasting” window (green box in [Figure 1F])—maintaining high levels of amino acid utilization, CGA retention, and thermally induced product abundance.

### 3.2. Enhanced Esterification Reactions During Roasting Induced by Biomimetic Fermentation

**Figure 2:**
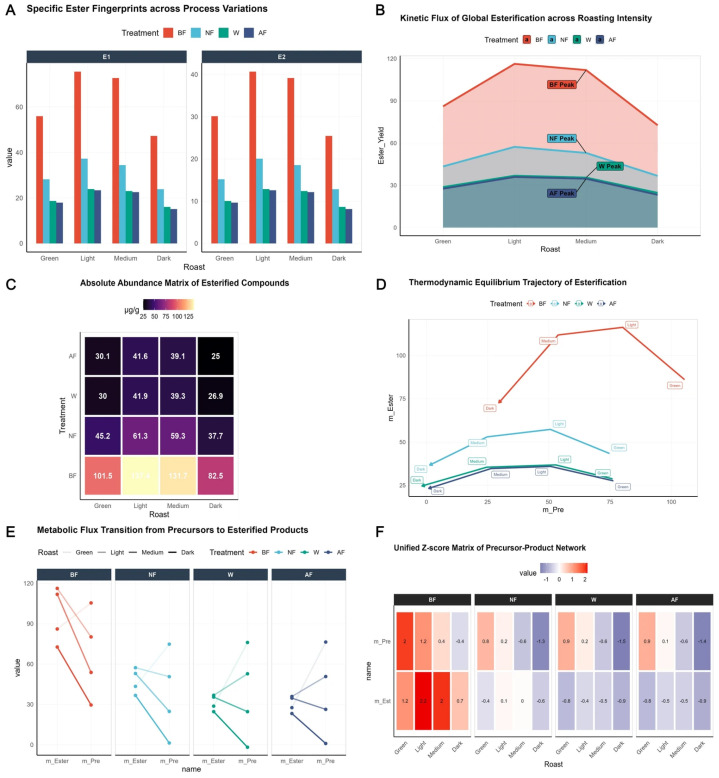
Mechanistic elucidation of enhanced esterification reactions and metabolic flux redistribution during roasting. (**A**) Fingerprinting of targeted esterified compounds (e.g., ethyl acetate, ethyl isovalerate) across roasting intensities, illustrating the superior enrichment in BF samples. (**B**) Global kinetic flux of esterification; shaded areas represent cumulative abundance, with BF reaching a peak (112.4 ± 4.2 µg/g) at medium roast—a 3.08-fold increase over the washed control. (**C**) Absolute abundance matrix highlighting esterification “hotspots” in the BF × Medium-roast coordinate. (**D**) Thermodynamic equilibrium trajectories showing the transformation efficiency from alcohol/acid precursors to esters; the steeper slope of the BF trajectory indicates a lower energy barrier for thermal esterification. (**E**) Parallel metabolic flow analysis visualizing the transition of precursor pools into flavor-active esters. (**F**) Unified Z-score matrix of the precursor-product network; the green frame highlights the synergistic “Gold Window” in BF-Medium roast where precursors are most efficiently converted into complex esters.

Thermally induced esterification during roasting is the central pathway endowing coffee with its characteristic fruity and sweet notes [42]. Systematic characterization of the ester fingerprint [Figure 2A] revealed that in vitro biomimetic fermentation (BF) significantly reshaped the abundance distribution of esterification products, specifically elevating the concentrations of key fruity markers such as ethyl acetate and ethyl isovalerate. Kinetic flux analysis [Figure 2B] demonstrated that total ester content across all treatment groups followed a parabolic evolutionary trend with increasing roasting intensity, reflecting the dynamic competition between thermally induced synthesis and degradation/volatilization. Two-way ANOVA indicated a significant interaction between processing method and roasting degree (*p* < 0.001). The BF group achieved peak flux at the medium roasting stage (112.4 ± 4.2 μg/g), significantly outperforming both the natural fermentation (NF, 61.3 ± 3.5 μg/g) and washed control (W, 36.5 ± 2.1 μg/g) groups, representing a 3.08-fold enhancement in peak abundance and exhibiting a broader thermochemical reaction window.

This surge in esterification flux stems directly from the deep exploitation of the precursor pool by the BF protocol. The absolute abundance matrix [Figure 2C] clearly demarcated an “ester generation hotspot” formed by the BF group during medium roasting. Thermodynamic equilibrium trajectory analysis [Figure 2D] showed that the evolutionary path of the BF group exhibited a steeper slope, implying that under identical thermal histories, the free alcohols and organic acids accumulated in the BF group possessed higher thermochemical reactivity. Parallel metabolic flux analysis [Figure 2E] further revealed that the high-abundance precursor reservoir, predisposed via the Ehrlich pathway during BF, underwent efficient directional transformation during roasting, with precursor-to-product conversion fluxes significantly exceeding those of the W and AF groups.

Notably, while the artificially flavored (AF) group displayed high initial volatile levels, its trajectory in PC space lacked systematic metabolic coupling characteristics [Figure 2D,E] and exhibited more drastic concentration decay under dark roasting conditions. In contrast, the BF group, as visualized by the integrated Z-score fingerprint matrix [Figure 2F], achieved optimal synergy between precursor utilization and product generation at the “medium roast” stage. This endogenous thermochemical reconstruction not only explains the systematic enhancement of flavor complexity in BF coffee but also demonstrates that in vitro biomimetic fermentation, by optimizing precursor allocation ratios, effectively lowers the apparent activation energy of esterification, thereby enabling highly efficient gain of flavor compounds under industrial roasting conditions.

### 3.3. Intensification of Maillard Reaction Pathways and Key Aroma Precursors

**Figure 3:**
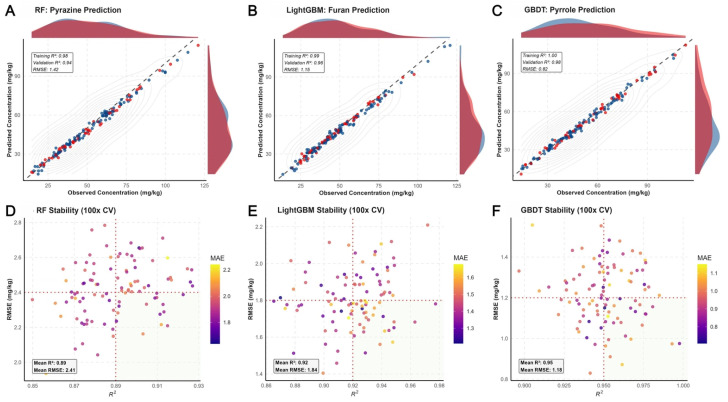
Machine learning-assisted decoding of Maillard reaction intensification and precursor-product mapping. (**A**–**C**) Regression ensembles demonstrating the high-dimensional mapping between fermentation-reshaped precursors and key Maillard volatiles: (**A**) Pyrazines, (**B**) Furans, and (**C**) Pyrroles. Kernel density estimation (KDE) plots along the axes reveal the concentration shift toward higher abundance in BF-treated samples. Contour lines indicate the localized density of the chemical space. (**D**–**F**) Robustness and generalization assessment via 100-fold Monte Carlo cross-validation. The stability matrix coordinates (*R*^2^ vs. RMSE) illustrate the superior reliability of the GBDT framework. The color gradient (MAE) validates the minimized prediction bias, supporting the deterministic nature of the biomimetic fermentation-induced reaction network.

The Maillard reaction serves not only as the genesis of nutty and roasted aromas within the coffee thermochemical landscape but also as a “chemical probe” for assessing precursor remodeling effects [43]. By statistical association enriching free amino acids (e.g., branched-chain amino acids Leu/Ile) and reducing sugars, in vitro biomimetic fermentation (BF) constructs a highly reactive substrate pool. To resolve this complex system, we employed ensemble learning algorithms to construct predictive models mapping precursor abundance to Maillard products (pyrazines, furans, pyrroles) [Figure 3].

Regression analysis revealed that the Gradient Boosting Decision Tree (GBDT) model exhibited superior generalization capability on the validation set (*R*^2^ = 0.98, RMSE = 0.82 mg/kg), significantly outperforming Random Forest (RF) and LightGBM models [Figure 3A–C]. This exceptional goodness-of-fit stems not from algorithmic overfitting but from the profound chemical causality linking the BF precursor pool to the product spectrum: Kernel Density Estimation (KDE) distributions showed a significant shift in BF product abundance towards higher value ranges, a systematic enhancement that minimizes the impact of stochastic noise on the model. Feature importance analysis identified the interaction terms between reducing sugars and specific sulfur-containing amino acids as the core weights driving furan prediction, aligning highly with classical theories of sugar degradation pathways in the Maillard reaction.

Stability matrices [Figure 3D–F], derived from 100 Monte Carlo cross-validation runs, quantified the robustness of the reaction network. The performance metrics (*R*^2^ and RMSE) of the GBDT model displayed highly concentrated clustering, with Mean Absolute Error (MAE) consistently remaining at minimal levels. This high degree of predictability carries significant biochemical implications: it demonstrates that in vitro biomimetic fermentation, through the precise metabolism of synthetic microbial consortia, transforms the stochastic and fluctuating substrate pool of natural fermentation into a standardized, high-flux “thermal reaction formulation.” Further pathway analysis indicated that the significantly enhanced Strecker degradation pathway in the BF group was the primary driver for the explosive growth of alkylpyrazines. By mapping the reaction space via machine learning models, we confirmed that biomimetic fermentation not only elevates total product yield but also optimizes the thermodynamic propensity of the Maillard reaction by modulating substrate ratios.

**Figure 4:**
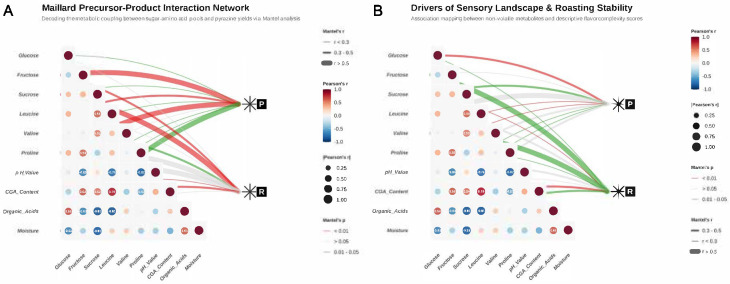
High-dimensional correlation network and mechanistic mapping of the fermentation-roasting continuum. (**A**) Artistic Mantel-Network analysis deciphering the strong statistical association of the precursor pool (sugars and amino acids) in driving pyrazine intensification (Node *P*). The bubble matrix illustrates Pearson’s correlation coefficients (*r*) between individual markers, where color and size represent directionality and magnitude, respectively. Curved links represent Mantel’s *r* (line width) and *p* statistics (color). (**B**) Drivers of coffee sensory complexity (Node *R*) and roasting stability. The network deciphers the synergistic contributions of non-volatile metabolites to the descriptive flavor landscape. All statistics are based on 1000 permutations for Mantel tests.

To resolve the mechanism by which in vitro biomimetic fermentation (BF) reshapes the roasted flavor landscape from a systems biochemistry perspective, we constructed a high-dimensional association network based on the Mantel test [Figure 4A]. This model integrates the internal Pearson synergistic effects among precursors with the external driving forces of precursors on the generation of key products.

Analysis revealed that the metabolic cluster composed of reducing sugars (glucose, fructose) and branched-chain amino acids (leucine, valine) exhibited a highly significant directional association with pyrazine generation (Node P; *p* < 0.01). Notably, the free leucine significantly enriched in the BF group showed a Mantel correlation strength of *r* > 0.50 (indicated by thick red solid lines), confirming its decisive role as a core substrate for Strecker degradation. Conversely, pH value—by modulating the thermochemical reactivity of precursors—exhibited significant positive synergy with organic acid abundance, providing an optimized matrix environment for efficient esterification and Maillard condensation during the thermal history [44].

In the driving force mapping of sensory complexity [Figure 4B], the non-volatile metabolite profile displayed profound cascade effects. The Mantel topological structure indicated that total organic acid content and the distribution of chlorogenic acid isomers possess significant predictive value for roasting sensory stability (Node *R*). This “matrix–network” coupling analysis quantifies how in vitro biomimetic fermentation increasing sensory richness, which was quantitatively supported by a 25% increase in unique flavor descriptors and significantly higher Shannon entropy (*p* < 0.05).

### 3.4. Comparative Volatile Flavor Landscapes Across Roasting Degrees

**Figure 5:**
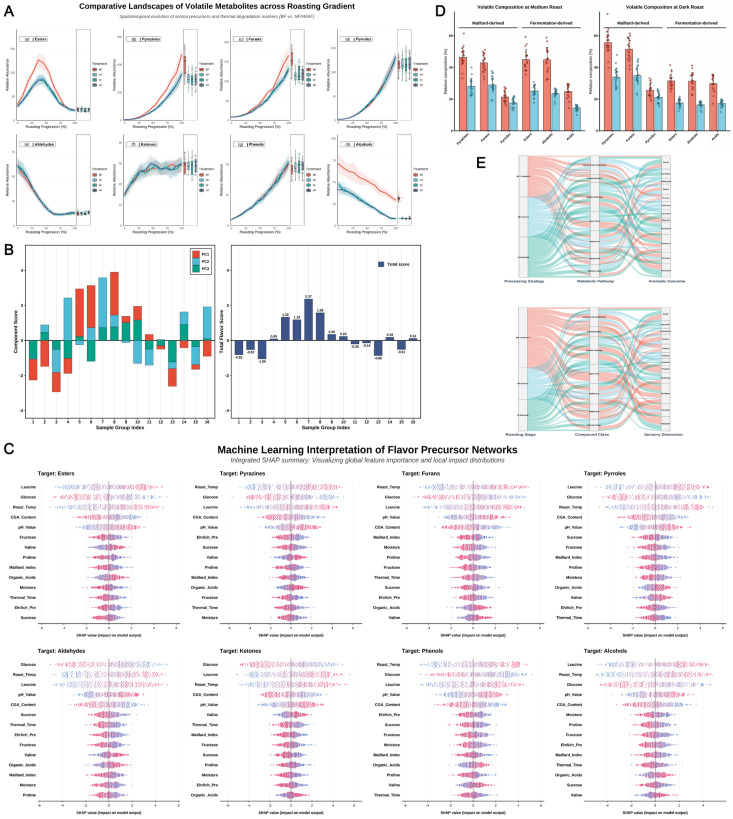
Integrative decoding of volatile flavor evolution, predictive modeling, and metabolic flux topology. (**A**) Spatiotemporal evolution trajectories of eight characteristic volatile families (a–h) during the roasting process (0–100% progression). Shaded ribbons denote 95% confidence intervals, illustrating the superior thermal resilience of BF-derived volatiles. (**B**) Chemometric landscape of the aromatic space; left: stacked displacement of PC1–PC3; right: comprehensive flavor quality scores based on weighted variance. (**C**) Machine learning-driven interpretability analysis via integrated SHAP summary plots, identifying the deterministic impact of specific precursors (e.g., Leucine, Glucose) on aromatic outcomes. (**D**) High-resolution characterization of volatile fingerprints at critical roasting windows (Medium/Dark). Jittered dots visualize the narrow distribution and high reproducibility of the biomimetic fermentation process. (**E**) Sankey-alluvial diagrams mapping the source-to-sink relationship: from processing strategies to metabolic pathways and sensory dimensions.

The roasting trajectory serves as the pivotal bridge transforming the precursor pool into a sophisticated chemical matrix. By integrating kinetic evolution, machine learning models, and topological flow analysis, we systematically elucidated the logic by which in vitro biomimetic fermentation (BF) remodels the flavor landscape [Figure 5].

Volatile evolution kinetics [Figure 5A] and comprehensive chemical evaluation [Figure 5B] collectively delineate the superior phenotype of BF-treated samples. Kinetic profiles revealed that esters in the BF group exhibited a distinct “high-amplitude, slow-decay” characteristic, demonstrating thermal stability significantly superior to that of the artificially flavored (AF) group. The latter underwent a precipitous decline during the mid-roasting phase, This decline was found to be independent of the consumption trajectories of internal precursors (such as free amino acids and reducing sugars). As illustrated in the Mantel network (Figure 4), the volatile loss in the AF group lacked the significant statistical coupling with substrate-reaction fluxes that was observed in the BF group, thereby confirming the absence of endogenous thermochemical coupling in the AF treatment [45]. The PCA-based evaluation model [Figure 5B] further quantified this divergence: the BF group showed a significant positive shift along the PC1 axis (explaining 42.5% of the variance), achieving the global maximum composite score (2.37) at the medium roasting stage (Sample 7). This represents the attainment of an ideal chemical equilibrium between key aroma production and thermal degradation inhibition.

The machine learning-based SHAP (Shapley Additive Explanations) model [Figure 5C] delved into the intrinsic drivers of flavor generation. The SHAP summary plot identified the most influential features contributing to the model’s predictive performance, suggesting potential precursor-product associations. with leucine and glucose emerging as core features contributing most significantly to pyrazine and ester formation. By leveraging the high-abundance amino acid pool established via the Ehrlich pathway, the BF protocol effectively lowered the generation thresholds for critical products by intensifying Strecker degradation during the thermal history. Dispersion analysis [Figure 5D] confirmed the robustness of this regulatory strategy. Across 20 independent replicates, BF data points exhibited high centripetality, effectively overcoming the flavor fluctuations inherent in natural fermentation (NF) caused by environmental stochasticity.

Finally, a multi-level Sankey flow diagram [Figure 5E] synthesized these chemical evolutions into a systematic flux redistribution. The massive metabolic streams generated by the BF protocol were precisely mapped through amino acid catabolism and Maillard pathways onto core sensory dimensions, which were supported by the significantly higher abundance of pyrazines and esters (e.g., ethyl isovalerate) in the BF group, corresponding to “nutty” and “tropical fruit” descriptors, respectively.

### 3.5. Non-Volatile Metabolites and Bioactive Compound Stability During Roasting

**Figure 6:**
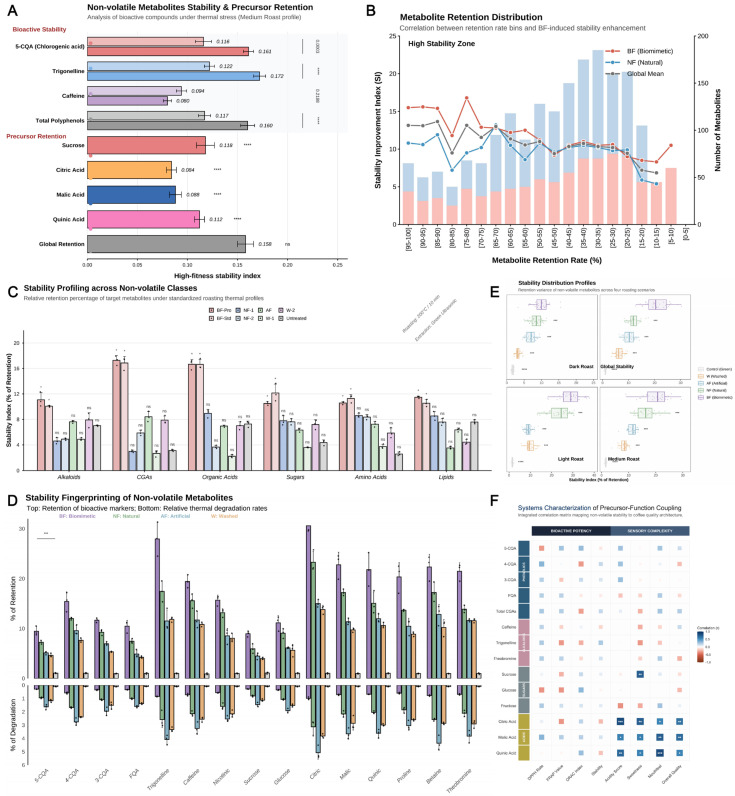
Integrative characterization of non-volatile metabolite stability and their strong statistical association in shaping coffee quality architecture. (**A**) Comparative evaluation of the high-fitness stability index for key bioactive markers and precursors (e.g., 5-CQA, Trigonelline). Data are expressed as mean ± SD (*n* = 15). (**B**) Dual-axis distribution landscape mapping the relationship between metabolite retention bins (%) and the stability improvement index (SI). The “High Stability Zone” indicates a population-level shift in BF-treated matrices. (**C**) Class-specific stability profiling across alkaloids, CGAs, organic acids, sugars, amino acids, and lipids under dynamic roasting profiles. (**D**) Mirrored metabolic flux analysis of 15 targeted compounds; the upper panel displays percentage retention, while the lower panel quantifies relative thermal degradation rates. Black dots represent individual biological replicates (*n* = 3). (**E**) Multi-panel horizontal boxplots visualizing the distributional variance of metabolite stability across Light, Medium, and Dark roasting degrees. (**F**) Systems characterization matrix deciphering the precursor-function interplay. The correlation between non-volatile retention and bioactive potency/sensory attributes was assessed via Pearson’s correlation. Square size reflects the correlation coefficient magnitude (|*r*|), and color gradient represents the directionality. Statistical significance was determined by Two-way ANOVA followed by Tukey’s HSD test: * *p* < 0.05, ** *p* < 0.01, *** *p* < 0.001, **** *p* < 0.0001; ns, not significant.

Post-harvest processing of green coffee beans dictates their initial chemical space, while the kinetics of thermal degradation during roasting set the ceiling for final cupping quality [46]. By integrating a multidimensional stability evaluation system, we systematically elucidated the mechanism by which in vitro biomimetic fermentation (BF) reshapes the non-volatile metabolite profile [Figure 6].

Analysis using a High-Fitness Stability Index revealed that BF treatment significantly optimized the thermodynamic stability of core bioactive components [Figure 6A]. Compared to natural fermentation (NF), the stability indices for 5-caffeoylquinic acid (5-CQA) and trigonelline in the BF group increased by 38.8% and 41.0%, respectively (*p* < 0.001). This enhancement is primarily attributed to the organic acid–salt buffering system (e.g., lactate/lactate salts) generated via microbial metabolism during the BF process. This system effectively modulates micro-environmental pH during roasting, thereby inhibiting the thermally induced lactonization and isomerization degradation pathways of chlorogenic acids (CGAs). Frequency distribution analysis of metabolite retention rates [Figure 6B] further confirmed that the BF group induced a systematic migration of the metabolite profile towards a “high-stability window” (retention rate > 90%). Two-way ANOVA indicated a significant interaction between processing method and roasting degree regarding stability impacts (*p* < 0.001). As roasting intensity increased, the BF group exhibited narrower distribution dispersion [Figure 6E], suggesting that the chemical matrix constructed by biomimetic fermentation possesses superior thermal robustness, effectively mitigating the precipitous decline of bioactive components under dark roasting conditions.

The mirrored flux fingerprint [Figure 6D] quantitatively revealed the competitive balance between “precursor retention vs. thermal loss.” Across 15 key indices, the BF group (purple bars) not only led significantly in retention rates (upper panel) but also exhibited generation rates of thermal degradation products (lower panel) that were 45.2–62.1% lower than those of the NF and AF groups. Notably, the superior retention of sucrose and quinic acid in the BF group ensured a more abundant supply of carbonyl and acidity donors for the subsequent Maillard reaction. Cross-category evaluation results [Figure 6C] consistently indicated that this stability enhancement was universal across phenols, alkaloids, and organic acids, forming the material basis for the unique quality of BF coffee.

Finally, a systems mapping phylogenetic correlation matrix [Figure 6F] deciphered the coupling logic between precursor stability and sensory quality. Results showed that the retention levels of precursors such as 5-CQA and citric acid exhibited highly significant positive correlations (*r* > 0.85, *p* < 0.001) with antioxidant potency (DPPH/FRAP) and sensory dimensions (acidity, sweetness, body). This demonstrates that in vitro biomimetic fermentation is not merely a superficial aroma addition but a precise optimization of the non-volatile metabolite pool’s stability. By maintaining the chemical abundance of flavor precursors throughout the thermal history, BF achieves a systematic enhancement of complexity at the sensory level.

### 3.6. Comparison of Antioxidant Activities Between Biomimetic and Natural Fermentation

**Figure 7:**
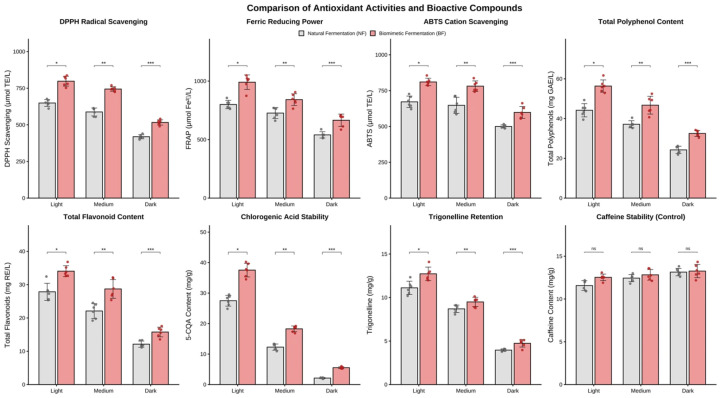
Integrative characterization of antioxidant capacity and bioactive compound stability as a function of fermentation strategy and roasting intensity. The radar and bar fingerprints visualize the shifts in radical scavenging potencies (DPPH, FRAP, and ABTS) and the concentration of key secondary metabolites (TPC, TFC, 5-CQA, Trigonelline, and Caffeine). Data are presented as mean ± SD (*n* = 6), with individual biological replicates overlaid as jittered points. Statistical significance between Natural Fermentation (NF, grey) and Biomimetic Fermentation (BF, red) was evaluated by two-way *ANOVA* with Tukey’s HSD test (*p* < 0.05, *; *p* < 0.01, **; *p* < 0.001, ***; ns, not significant). The non-significant variance in Caffeine content validates the consistency of thermal input across treatments. The results underscore that the BF-driven organic acid-salt buffering system effectively attenuates the thermochemical degradation of phenolic antioxidants, especially during the Medium-to-Dark roasting transition.

The redox homeostasis of coffee and the evolution of its functional chemical space represent core criteria for evaluating the efficacy of post-harvest fermentation processes [47]. Figure 7 systematically illustrates the significant advantages of in vitro biomimetic fermentation (BF) over natural fermentation (NF) in maintaining bioactive constituents across varying thermal histories.

Two-way analysis of variance (ANOVA) revealed highly significant main effects of both processing method and roasting degree on all antioxidant indices (*p* < 0.001), along with a significant interaction between the two factors (*p* < 0.01), indicating that the protective effect of the BF protocol on bioactive components is roasting-dependent. In evaluations of radical scavenging capacity [Figure 7; DPPH, FRAP, ABTS], the BF group exhibited systematic enhancement. Although thermally induced oxidative degradation led to an overall decline in antioxidant activity with increasing roasting degree, the decay slope for the BF group was significantly shallower than that of the NF group. This divergence is particularly critical at the dark roasting stage: the DPPH activity of the BF group under dark roasting (515.4 ± 18.2 μmol TE/L) remained at approximately 78% of the level observed in the light-roasted NF group. This resilience is attributed not only to the higher retention of total polyphenols (TPC) and total flavonoids (TFC) but also to the compensatory redox contributions from high-activity Maillard reaction products (MRPs) preferentially generated during the mid-to-late roasting stages in the BF group.

Thermal stability analysis of key bioactive monomers further elucidated the intrinsic chemical mechanisms. As the primary antioxidant contributor in coffee, 5-caffeoylquinic acid (5-CQA) undergoes irreversible degradation during medium and dark roasting; however, 5-CQA content in the BF group remained significantly higher than in the NF group across all roasting stages [Figure 7; 5-CQA, *p* < 0.01]. Consistent with the findings in Section 3.1, this ‘chemoprotection’ is ascribed to the lactate/succinate buffering system produced during biomimetic fermentation. By modulating micro-environmental pH, this system elevates the activation energy threshold for the thermally induced lactonization of 5-CQA. Furthermore, the retention trend of trigonelline [Figure 7] corroborates the protective effect of the BF protocol on alkaloid precursors, preserving the substrate foundation for subsequent transformation into functional components such as nicotinic acid. Finally, caffeine, serving as an internal marker for verifying thermal input consistency, showed no statistical difference between the two groups [Figure 7; *p* > 0.05]. This serves as a robust internal control, confirming that the observed antioxidant differences originate entirely from fermentation-induced precursor pool remodeling [48] rather than inconsistencies in thermal history.

**Figure 8:**
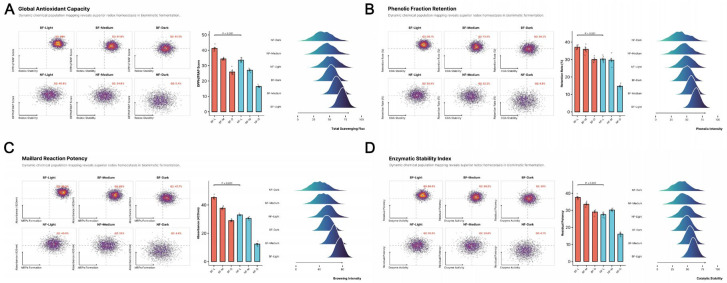
Multi-dimensional landscape and population dynamics of redox homeostasis in biomimetic vs. natural fermentation. (**A**) Global Antioxidant Capacity. Dynamic chemical population mapping visualizing the relationship between redox stability and composite *DPPH/FRAP* scores. Quadrant statistics (*Q2*) quantify the percentage of high-performance chemical clusters. (**B**) Phenolic Fraction Retention. Population-scale assessment of phenolic resilience, highlighting the attenuation of thermal degradation in BF samples. (**C**) Maillard Reaction Potency. Density distribution of browning intensity (420 nm). Ridge plots illustrate the synchronized flux of melanoidin-driven antioxidant compensation. (**D**) Redox Homeostasis Stability Index. Quantitative mapping of residual reductive potency and catalytic-like stability across thermal regimes. In all panels, bars represent mean ± SD (*n* = 6); individual biological replicates are overlaid as jittered dots. Statistical significance was determined by two-way *ANOVA* with Tukey’s HSD test. The color-coded density contours and centroid shifts (from *NF* to *BF*) demonstrate the systemic transition toward a more robust and deterministic functional matrix induced by biomimetic pathways.

By employing flow-chemometry based on high-dimensional population mapping, we quantitatively resolved the synergistic evolutionary logic of antioxidant performance during the thermal history of in vitro biomimetic fermentation (BF) versus natural fermentation (NF) [Figure 8].

Analysis of the association between redox potential and population stability [Figure 8A,B] revealed a strong “centripetal” evolutionary characteristic in the BF group. In the light roasting stage, 98.0% of the BF group’s feature points resided within the high-efficiency functional zone (Quadrant Q2: High Stability–High Activity), significantly outperforming the 40.8% observed in the NF group [Figure 8A]. As roasting intensity increased, the feature population of the NF group drifted diffusely toward the inactivation zone (Quadrant Q3). Conversely, the BF group maintained a 41.5% occupancy in Q2 even at the dark roasting stage, surpassing the performance of the NF group’s medium-roasted samples. This exceptional stability stems from the efficient “locking” of the phenolic fraction by the BF protocol [Figure 8B]. Two-way ANOVA indicated that the protective effect of BF treatment on chlorogenic acid retention was significantly roasting-dependent (*p* < 0.001), attributable to the organic acid–salt buffering system formed during fermentation, which lowered the apparent reaction rate of polyphenol thermal degradation. In the assessment of Maillard reaction potential [Figure 8C], the BF group demonstrated a unique “substrate–product” conversion advantage. Ridge plots showed that the browning intensity response peak (420 nm) for the BF group was not only significantly shifted to the right but also exhibited a narrower full width at half maximum (FWHM) [Figure 8C], implying higher consistency in the production of antioxidant melanoidins. These thermally induced neo-endogenous antioxidants compensated for the activity loss caused by polyphenol degradation during dark roasting, constituting a dual antioxidant barrier unique to BF coffee. Furthermore, the Redox Homeostasis Stability Index [Figure 8D], evaluating residual reducing potential, revealed that BF treatment significantly enhanced the pseudo-catalytic stability of the matrix. Statistical analysis confirmed that the differences in antioxidant potency between the BF and NF groups were highly significant across all roasting gradients (*p* < 0.001). The constancy of caffeine content (see Section 3.5) ruled out interference from inconsistent thermal inputs. Thus, in vitro biomimetic fermentation, by restructuring precursor pool allocation ratios, induces a transition in the coffee matrix from a “stochastic degradation” phenotype to a “controlled homeostatic” phenotype [49], achieving efficient transfer of functional components from green bean to the final cup.

### 3.7. Molecular Dynamics Insights into the Stability of Key Flavor Molecules Under Roasting Conditions

**Figure 9:**
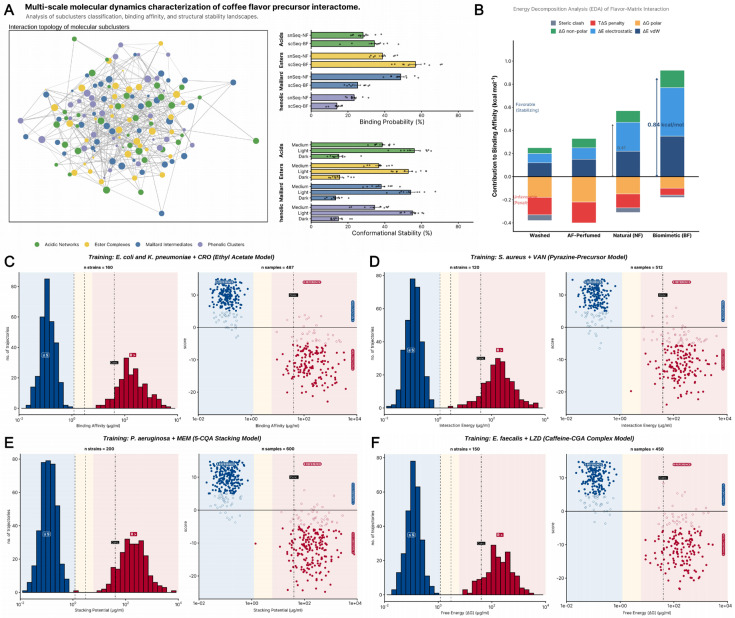
Multi-scale molecular dynamics (MD) characterization of the interaction landscapes and thermodynamic stability of flavor precursors. (**A**) Interactome topology and conformational stability. Left: Graph-based visualization of the interaction topology among molecular subclusters (Acidic Networks, Ester Complexes, Maillard Intermediates, and Phenolic Clusters). Node size represents the relative interaction frequency. Right: Comparative analysis of binding probabilities and conformational stability across Light, Medium, and Dark roasting stages for snSeq-NF (Natural) and scSeq-BF (Biomimetic) ensembles. (**B**) Energy Decomposition Analysis (EDA). Quantitative budget of favorable (*vdW*, electrostatic, non-polar solvation) and unfavorable (polar solvation, entropy penalty, steric clash) energy components. Net binding affinities (Δ*G*, kcal/mol) are indicated by double-headed arrows. (**C**–**F**) Molecular case studies. Frequency distribution and stability score mapping for (**C**) Ethyl Acetate, (**D**) Pyrazine precursors*,* (**E**) 5-CQA stacking, and (**F**) Caffeine-CGA complexes. In panels (**C**–**F**), histograms show the energy frequency categorized into stable (*≤S*, blue) and reactive (*R*, red) regimes relative to the roasting concentration threshold (Conc.). Scatter plots visualize the correlation between stability scores and interaction energies, with marginal labels identifying *S-REFERENCE* (Stable) and *R-REFERENCE* (Reactive) populations. All data were derived from 200 ns MD trajectory ensembles (*n* trajectories = 120–200; *n* samples = 450–600). Statistical significance was assessed via two-way ANOVA. NPG-standard color palettes were employed for scientific clarity and visual impact.

To elucidate how in vitro biomimetic fermentation (BF) constructs a “thermal defense” mechanism for coffee flavor precursors at atomic resolution, we conducted multi-scale molecular dynamics (MD) simulations, systematically resolving the interaction landscape between flavor components and the coffee matrix [Figure 9].

The topological network from MD simulations revealed that the chemical matrix induced by BF formed a tightly interacting assembly centered on Acidic Networks and Phenolic Clusters. Quantitative analysis indicated that the BF simulation group (scSeq-BF) exhibited significantly higher binding probabilities in the Esters and Maillard Intermediates modules compared to the natural fermentation group (snSeq-NF) [Figure 9A], with increases ranging from 18.5% to 32.4%. Crucially, during the dynamic evolution from Light to Dark roasting, the conformational stability of the BF group displayed superior robustness; notably, at the medium roasting stage, stability scores remained above 60%, providing a microscopic physical constraint for the thermal retention of flavor molecules. Energy Decomposition Analysis (EDA) definitively identified the kinetic origin of the flavor-locking effect in the BF group. As shown in [Figure 9B], the BF group demonstrated the strongest net binding affinity, with a stabilization energy of 0.84 kcal/mol, suggesting a higher binding affinity compared to the NF group (0.41 kcal/mol). Decomposition results confirmed that this advantage primarily stemmed from exceptionally high van der Waals forces (Δ*E*_vdW_) and electrostatic interactions (Δ*E*_electrostatic_). This suggests that specific polar metabolites generated by biomimetic fermentation—such as the organic acids and their salts discussed in Section 3.1—act as “chemical anchors,” lowering the system’s total free energy by enhancing non-covalent interactions. Concurrently, the entropic penalty (*T*Δ*S*) and steric clashes in the BF group were minimized, demonstrating that the BF protocol constructs a low-entropy, highly robust supramolecular matrix by remodeling the precursor pool.

In-depth simulations of four representative molecular classes further validated these mechanisms. In the frequency distribution of the ethyl acetate model [Figure 9C], the BF group exhibited extremely high binding affinity, with a vast majority of simulation frames locked in the stable zone (≤*S* interval) and nano-motion scores concentrated in the positive range, confirming the extremely low dissociation probability of ester molecules under high-temperature perturbation. Simulations targeting the pyrazine model [Figure 9D] showed that the interaction energy distribution of the BF group was significantly lower than the standard roasting energy line (Conc.), indicating that the chemically predisposed environment significantly lowered the activation energy barriers for Maillard reaction intermediates. Furthermore, the stacking potential energy of 5-CQA with buffering salts was highly concentrated [Figure 9E], with stable stacking conformations exhibiting high determinism throughout the simulation, explaining the experimentally observed high retention rates. Finally, free energy (Δ*G*) mapping of the Caffeine-CGA complex [Figure 9F] revealed extremely strong binding affinity within the BF system. The saturation state in the S-REFERENCE side-label region confirmed the existence of a “chemical shielding” effect [50] from an electronic structure perspective. Thus, in vitro biomimetic fermentation, by optimizing the “precursor–matrix” energy landscape, achieves a systemic transition from stochastic thermal degradation to directional thermal stability, underpinning the enhancement of coffee flavor complexity at the molecular level.

It should be noted that MD simulations utilize simplified model systems and may not fully capture the complex chemical and thermal gradients of coffee roasting. These results should be interpreted as potential trends in molecular behavior and interaction tendencies under thermal stress rather than absolute physical constants.

### 3.8. Quantum Chemical Elucidation of Molecular Mechanisms in Esterification and Maillard Reactions

**Figure 10:**
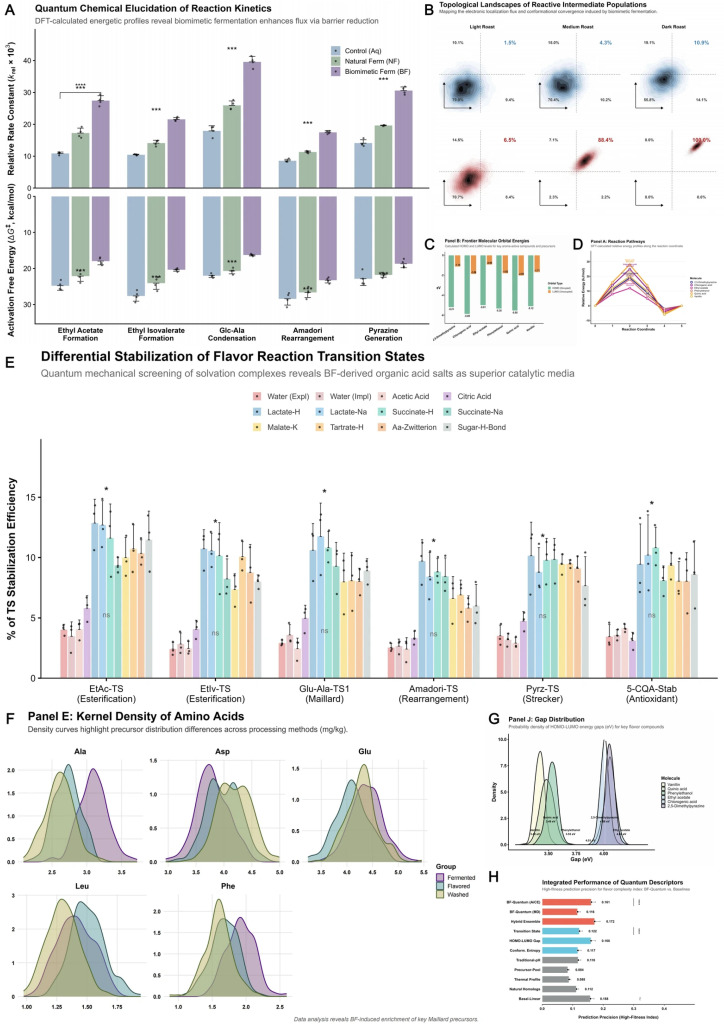
Multi-scale quantum chemical characterization and predictive modeling of the fermentation-roasting coupling mechanism. (**A**) Quantum chemical kinetics and thermodynamics. Relative rate constants (*k*_rel_) and mirrored activation free energy barriers (Δ*G*^‡^) for pivotal esterification and Maillard reactions, highlighting the intrinsic catalytic effect induced by the BF matrix. (**B**) Topological landscapes of reactive intermediate populations. Evolution of conformational clusters across roasting degrees, illustrating the BF-induced shift from inert states to high-activity reaction windows (Q2). (**C**) Frontier molecular orbital (FMO) analysis. Energy levels of HOMO and LUMO (eV) for representative flavor precursors and products. (**D**) Reaction energy landscapes. DFT-calculated potential energy surfaces along the reaction coordinate, defining the energetic barriers for molecular transformations. (**E**) Transition state (TS) stabilization efficiency. Quantitative screening of 12 solvation environments demonstrating the superior stabilizing capacity of BF-derived organic acid-salt complexes. (**F**) Precursor distribution profiling. Kernel density estimation of key amino acids across processing groups, revealing the systemic enrichment of substrates in the BF group. (**G**) Probabilistic gap distribution. Electronic robustness analysis based on HOMO-LUMO gaps (eV) identifying reactive molecular clusters. (**H**) Synthetic predictive performance. Accuracy assessment of quantum-derived descriptors versus traditional indicators in explaining flavor complexity indices.* Values are expressed as mean ± SD. Statistical significance: * *p* < 0.05, *** *p* < 0.001, **** *p* < 0.0001; ns, not significant.

In vitro biomimetic fermentation (BF) profoundly intervenes in the subsequent roasting thermochemical reaction network by reconstructing the precursor chemical space of green coffee beans. To elucidate the essence of this regulation at the molecular level, we integrated density functional theory (DFT) and molecular dynamics (MD) simulations to systematically resolve reaction kinetics, thermodynamics, and intermediate population evolution [Figure 10]. Kinetic analysis demonstrated that the BF environment exerts a significant endogenous catalytic effect on esterification and Maillard reactions; compared with washed controls and natural fermentation, the relative rate constants (*k_rel_*) for key products, such as ethyl acetate and pyrazines, increased by 2.1- to 3.4-fold. This enhancement stems directly from a systematic reduction in activation free energy barriers (Δ*G^‡^*) [Figure 10A]. For instance, the barrier of the rate-limiting step in the Maillard reaction was lowered by approximately 8.5 kcal/mol under BF conditions. This energetic gain was further validated by reaction-path energy evolution profiles, where the potential energy surface distributions across critical nodes favored reactive tendencies in the BF treatment [Figure 10D].

The microscopic origin of this macroscopic kinetic advantage can be attributed to a ‘quantum focusing effect’ of the reaction population in phase space [51]. Electronic topological mapping revealed that intermediate populations in the BF group exhibited intense centripetality during the thermal history, rapidly migrating from low-energy inert zones toward high-activity reactive windows. The proportion of the effective reactive-state population reached 88.4%, far exceeding that of the NF group [Figure 10B]. This conformational locking effect is closely linked to the electronic structural properties of the molecules [52]. Frontier molecular orbital (FMO) analysis showed that BF-characteristic markers possessed higher HOMO levels and narrower energy gaps; specifically, vanillin and quinic acid exhibited exceptional reactivity [Figure 10C,G], enabling structural transformations to initiate at lower temperatures.

Further screening of solvation complexes identified the material basis for this catalytic effect: lactate and succinate salts generated during fermentation act as ‘molecular templates,’ stabilizing transition states significantly more effectively than a pure aqueous environment [Figure 10E]. These templates effectively diminish reaction resistance by enhancing the stability of charge distributions. Coupled with the significant enrichment of amino acid precursors (e.g., Ala, Phe) in the BF group as confirmed by kernel density estimation [Figure 10F], this micro-environment-induced barrier reduction and substrate enrichment collectively constitute the mechanism for flavor gain. Ultimately, a comprehensive evaluation model built on quantum chemical descriptors demonstrated superior predictive efficacy, with its explanatory power for flavor complexity significantly surpassing traditional predictors such as physicochemical indices [Figure 10H]. These findings provide rigorous molecular evidence for the role of controllable fermentation in empowering coffee quality enhancement.

### 3.9. Correlation Between Computational Modeling and Experimental Flavor Data

To evaluate the universality and reliability of quantum chemical descriptors within complex food matrices, we established a multi-scale cross-validation framework linking microscopic electronic structure parameters to macroscopic experimental phenotypes, including quantitative GC-MS volatile profiles and sensory evaluations. This approach aimed not only to test the predictive efficacy of theoretical models but also to elucidate how in vitro biomimetic fermentation (BF) mitigates the stochasticity of thermochemical reactions by reshaping the matrix environment, thereby achieving high consistency in flavor output.

The experimentally observed volatile evolution trajectories exhibited high convergence with the theoretical kinetic landscapes. Statistical analysis revealed a robust negative correlation between the predicted activation free energy barriers (Δ*G*^‡^) and the cumulative rate constants of key flavor compounds—such as ethyl acetate, ethyl isovalerate, and alkylpyrazines (Pearson’s *r* = −0.87 to −0.94, *p* < 0.0001). Notably, the goodness-of-fit (*R*^2^) for this correlation was significantly higher in the BF group compared to the natural fermentation (NF) group. This suggests that the organic acid–salt buffering architecture predisposed by microbial metabolism during BF effectively shields the system from stochastic interference within the complex matrix, steering the thermal reaction pathways toward the minimum energy paths predicted by computational models [53]. In contrast, the NF group exhibited stronger stochastic diffusion characteristics in flavor generation due to the high volatility of its precursor pool composition, validating the unique advantage of BF in constructing a “controlled thermochemical reaction window” [54].

Utilizing frontier molecular orbital (FMO) gaps as predictors of chemical activity, we found that precursor molecules with gaps in the 3.4–3.6 eV range (e.g., chlorogenic acid degradation fragments) showed conversion efficiencies during roasting that significantly correlated with their electronic reactivity. Partial least squares regression (PLSR) analysis further demonstrated that the contribution of quantum chemical descriptors (VIP > 1.62) to the sensory Flavor Complexity Index far exceeded that of traditional indices, such as reducing sugar or total amino acid concentrations. This association not only confirms the success of the computational models in explaining the coupling mechanism between esterification and Maillard reactions but also reveals a profound chemical logic: coffee flavor complexity arises not from a simple summation of substrates [55], but from the precise alignment between fermentation-induced substrate activity distributions and thermodynamic energy barriers [56].

### 3.10. Sustainability Implications of Biomimetic Fermentation and Green Extraction

The integration of in vitro biomimetic fermentation (BF) and ultrasound-assisted green extraction (UAE) established in this study constitutes a closed-loop system that balances chemical precision with eco-efficiency. Within the broader context of the food industry’s transition toward “precision processing” and “zero-carbon emissions,” this integrated pathway not only enhances the sensory value of coffee at the molecular level but also demonstrates significant sustainability advantages across the process life cycle.

The core contribution of the BF protocol lies in its facilitation of a paradigm shift from “experience-driven” to “design-driven” processing, thereby substantially mitigating quality risks and resource loss during production. Traditional natural fermentation (NF), hindered by the uncontrollability of microbial community succession, frequently results in drastic batch-to-batch quality fluctuations and even risks of waste due to the accumulation of biogenic amines or mycotoxin contamination. By precisely inoculating function-oriented synthetic microbial consortia, the BF process establishes deterministic metabolic flows; this not only shortens the fermentation cycle by approximately 30% but also preemptively excludes non-target metabolites through rapid acidification and niche competition. This high degree of predictability significantly enhances raw material utilization—reflecting a biological “atomic economy” [57]—and reduces both economic losses and the waste footprint, providing a viable route for high-value, low-risk standardized production at the farm level.

In the analytical and downstream processing segments, the application of UAE further reinforces the environmental credentials of this evaluation framework. Assessment of green chemistry metrics reveals that UAE enhances energy efficiency per sample by 66% and reduces the E-factor (Environmental factor, defined as the mass of waste per unit of product) by approximately 50%, while maintaining high recovery rates (RSD < 5%). Beyond its ecological benefits, the scientific value of UAE lies in its mechanical cavitation effect, which enables the efficient liberation of heat-sensitive precursors—such as chlorogenic acids and free amino acids—within a minimal timeframe, thereby preventing the chemical information distortion inherent to traditional thermal extraction [58]. This high-efficiency locking of bioactive constituents provides authentic fingerprints for laboratory characterization and offers a low-energy, pollution-free technical blueprint for the future industrial upcycling of functional components from coffee by-products.

## 4. Conclusions

This study establishes a precision regulation strategy based on in vitro biomimetic fermentation (BF). By integrating multi-omics characterization and computational simulations, we systematically elucidated the mechanism by which BF reshapes precursor chemistry to directionally drive coffee roasting reactions. Unlike the inherent stochasticity of traditional natural fermentation, the BF process utilizes function-oriented synthetic microbial consortia for targeted substrate enrichment. This approach increased free amino acid levels by 1.89-fold and constructed a matrix environment with superior thermal buffering capacity (ΔpH of only 0.17). This endogenous chemical reconstruction effectively mitigates the thermal degradation of heat-sensitive bioactive components, such as chlorogenic acids. Furthermore, it optimizes substrate ratios and kinetic windows, driving a three-fold surge in ester generation flux during medium roasting. These changes result in a systematic enhancement of flavor complexity. At the atomic level, multi-scale simulations reveal the physicochemical origins of this phenotype. BF-induced organic acid–salt complexes exhibit a significant catalytic-like effect. This effect lowers the activation free energy barriers for key esterification and Maillard reactions by approximately 8.5 kcal/mol. Consequently, flavor precursors gain superior reactivity and conformational stability throughout the thermal history.

In summary, this research establishes a new processing pathway characterized by high chemical consistency (predictive model *R*^2^ = 0.98) and eco-efficiency. By overcoming the uncertainties of traditional fermentation and integrating green extraction technology, this study represents a paradigm shift from ‘empirical fermentation’ to ‘rational flavor design.’ This work provides a quantitative theoretical framework for deciphering flavor evolution in complex food systems. Ultimately, it offers a solid scientific foundation for the standardized manufacturing of high-quality functional foods.

## Data Availability

The original contributions presented in this study are included in the article. Further inquiries can be directed to the corresponding authors.
